# Genotyping of KRAS Mutational Status by the In-Check Lab-on-Chip Platform

**DOI:** 10.3390/s18010131

**Published:** 2018-01-05

**Authors:** Maria Guarnaccia, Rosario Iemmolo, Floriana San Biagio, Enrico Alessi, Sebastiano Cavallaro

**Affiliations:** 1 Institute of Neurological Sciences, Italian National Research Council, Via Paolo Gaifami 18, 95126 Catania, Italy; maria.guarnaccia@cnr.it (M.G); iemmolo.rosario@gmail.com (R.I.); 2 STMicroelectronics, Stradale Primosole 50, 95121 Catania, Italy; floriana.sanbiagio@st.com; 3 Analog, MEMS & Sensor Group—HealthCare Business Development Unit, STMicroelectronics, Stradale Primosole 50, 95121 Catania, Italy; enrico.alessi@st.com

**Keywords:** Lab-on-Chip, PCR, Microarray, nucleic acids analysis, KRAS, diagnostic

## Abstract

The KRAS oncogene is involved in the pathogenesis of several types of cancer, particularly colorectal cancer (CRC). The most frequent mutations in this gene are associated with poor survival, increased tumor aggressiveness and resistance to therapy with anti-epidermal growth factor receptor (EGFR) antibodies. For this reason, KRAS mutation testing has become increasingly common in clinical practice for personalized cancer treatments of CRC patients. Detection methods for KRAS mutations are currently expensive, laborious, time-consuming and often lack of diagnostic sensitivity and specificity. In this study, we describe the development of a Lab-on-Chip assay for genotyping of KRAS mutational status. This assay, based on the In-Check platform, integrates microfluidic handling, a multiplex polymerase chain reaction (PCR) and a low-density microarray. This integrated sample-to-result system enables the detection of KRAS point mutations, including those occurring in codons 12 and 13 of exon 2, 59 and 61 of exon 3, 117 and 146 of exon 4. Thanks to its miniaturization, automation, rapid analysis, minimal risk of sample contamination, increased accuracy and reproducibility of results, this Lab-on-Chip platform may offer immediate opportunities to simplify KRAS genotyping into clinical routine.

## 1. Introduction

KRAS belongs to a group of genes encoding small GTP-binding proteins, known as the RAS superfamily or RAS-like GTPases. In different human tumor types, such as bowel subtypes (cecum, ascending and descending colon, splenic flexure, transverse colon) and sporadic CRC, with the extreme example of pancreatic and lungs cancer, the mutated KRAS acquires oncogenic properties acting as a mediator in cancer development and growth [1,2,3,4]. In patients affected with bowel disease-associated colon cancer, KRAS mutations are associated with high frequency with the progression of benign adenoma in a dysplastic adenocarcinoma and distant metastases [5,6]. The most frequent mutations in KRAS involve single nucleotide substitutions, occurring in codons 12 and 13 of exon 2 or, less frequently, in codons 61 (exon 3) and 146 (exon 4). The KRAS protein, also called p21, is a membrane-anchored guanosine triphosphate/guanosine diphosphate (GTP/GDP)-binding protein widely expressed in most human cells. Thanks to its GTP hydrolytic activity, KRAS switches between an active GTP-bound form to an inactive GDP-bound status [7]. Substitution of glycine with another amino acid occurring in codon 12 (G12D, G12A, G12R, G12C, G12S, G12V) or 13 (G13D), disrupts the GTPase activity of KRAS protein that turns into a constitutively active state [8]. Recruitment of an active KRAS to the membrane stimulates the activation of multiple downstream proliferative signaling pathways, such as the mitogen-activated protein kinase (MAPK), RAF/MEK/ERK, PI3K/AKT, and the RAL/GDS signaling cascades [9,10]. These signaling pathways affect multiple cellular processes that are critical for tumor progression, proliferation and survival, invasion and metastasis, cell polarity and movement, as well as for drug therapy outcomes [11]. In this regard, a significant association exists between molecular alterations of KRAS and resistance to targeted therapies. The gain-of-function KRAS mutations, in particular, are responsible for bypassing EGFR upstream signals resulting in poor response to anti-EGFR therapies in 30–40% of colorectal cancer [12,13]. For these reasons, the National Comprehensive Cancer Network (NCCN) and the American Society for Clinical Oncology (ASCO) recommend KRAS mutational test for patients with metastatic CRC, who are candidates for anti-EGFR treatment [5]. Knowledge of KRAS mutation status, therefore, is nowadays used to predict which patients are responsive to anti-EGFR therapies [14]. An early detection of KRAS mutation, moreover, can prevent the development of long-term complications, allowing a relevant advancement towards a more personalized and cost-effective medicine [15].

Currently, detection methods of KRAS mutations are based on direct sequencing (dideoxy and pyrosequencing), hybridization or amplification technologies, such as high-resolution melting analysis (HRMA), amplification refractory mutation system (ARMS), and cleaved amplification polymorphism sequence-tagged sites (PCR-RFLP) [16,17]. The complexity, long-time for results, requirement of highly skilled personnel, sophisticated equipment and complex bioinformatics make these methods less suitable for the clinical practice [3,18]. In addition, they require expensive procedures and high costs for single mutation detection with clear disadvantages for Public Healthcare. The actual challenge, therefore, is the adoption of new diagnostic strategies, more sensitive, easy to use, with lower cost, increased accuracy and reproducibility of results [19,20].

The In-Check platform developed by STMicroelectronics is a miniaturized Lab-on-Chip (LOC) device, brings together silicon-based microelectronics with micromachining technology, allowing a fast, highly sensitive and specific analysis of nucleic acids. This technology, in particular, integrates PCR and low-density microarray modules on a miniaturized silicon chip through a fluidic bypass, as reported elsewhere [21]. Amplification of nucleic acids and competitive hybridization of generated amplicons were performed simultaneously [18,22,23]. The opportunity to reproduce a PCR-Microarray integrated method on a chip, miniaturizing the entire processes, permits to combine the advantages of these technologies, in term of high sensitivity, simplicity and high-throughput, with rapid heating/cooling rates, short assay time, low reagent consumption, and easiness to automation [19,24,25]. In particular, the PCR module allows fast temperature ramping with an accuracy of 0.1 °C, improving performance and amplification time. The miniaturized low density microarray device, comprising 126 spots, offers the opportunity to design and customize the chip, suiting individual applications [26]. In addition to PCR and microarray modules, the main components of the In-Check platform are the Temperature Control System (TCS) that actuates, monitors, and controls the parameters of the reactions, together with the portable Optical Reader (OR) that scans the chip, and the software that controls the instruments and runs the data analysis [18,23,27]. 

In this study, we describe the development of a Lab-on-Chip assay, based on the In-Check platform, for detection of KRAS mutations in codons 12, 13, 59, 61, 117, and 146.

## 2. Materials and Methods

### 2.1. Assay Design

To assess the cancer mutations profiling of KRAS, we performed the following preliminary steps: (i) identification of the KRAS point mutation panel, (ii) design of PCR primers to detect target sequences of interest, (iii) probe set design for the chip array. 

**Figure 1:**
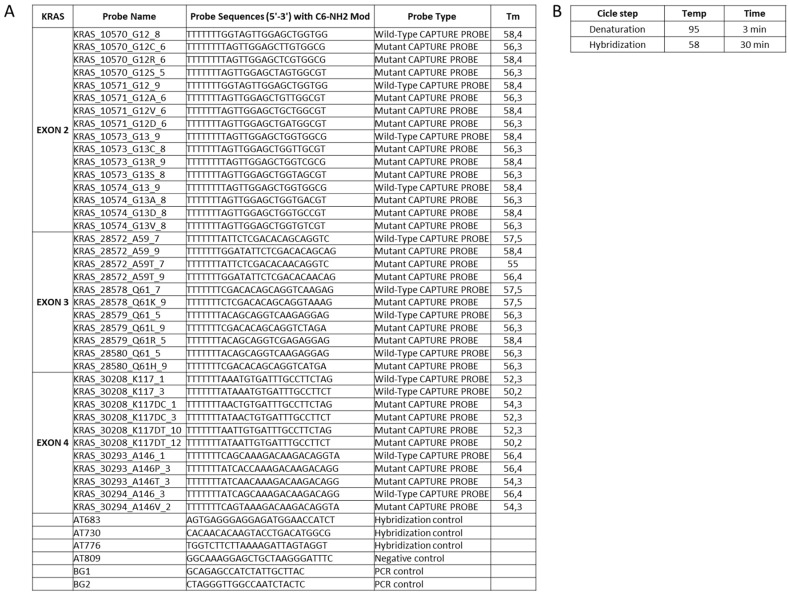
Probes set designed to identify the SNP of interest (**A**) and the protocol for the competitive hybridization step on the chip array (**B**); on the chip array surface was also spotted the capture probes used for hybridization, negative and positive control.

(i) The KRAS point mutation panel was identified by the use of public database annotations including the most commonly observed single nucleotide variations/polymorphisms (SNPs). The selection criteria were based on population frequency data and their involvement to CRC cancer (Table 1). 

(ii) In order to obtain the sequence of interest, we used the automated design tool (Primer-Blast) to select appropriate flanking primers and assess the parameters for PCR amplification [28]. Two primers were considered for each SNP, and since the microarray probes were designed to capture the reverse strand of the PCR product, the reverse primers were labeled with Cy5 at its 5′ end (Table 2). All the primers selected were used to amplify the genomic DNA, and PCR products were detected by electrophoresis on 1.5% agarose gel.

(iii) In a hybridization-based method, the probes design is a crucial step: the efficiency depends on a strong affinity between the specific target and the short oligonucleotide probes [29,30]. The probe set selection strategy was mainly based on sequence similarity threshold, thermodynamic properties, sensitivity and specificity, the range of GC content for uniform hybridization reaction, cross-hybridization efficiency and the accuracy for the detection of KRAS polymorphisms (Figure 1) [31]. The probes set effectiveness were evaluated using a dedicated software. In the low density array chip, the number of spotted probes can vary from 126 to 400. In our layout, were immobilized 114 probes including two replicate spots of hybridization control probes, mutated and wild-type probes to improve statistical significance of results (see below).

### 2.2. Samples Processing

The sample to results workflow takes less than 2 hr and includes the following steps: (1) extraction of total high quality DNA from tissues, (2) PCR amplification of target sequences on the chip chambers, (3) hybridization on the chip array surface, (4) post-hybridization washing, (5) scanning of the chip array by OR module at the specific probe wavelength, (6) image acquisition and data analysis using an imaging software.

The genomic DNA was extracted from paraffin-embedded tissue sample by use of a fully automated method; the quality and quantity parameters of all DNA samples were estimates using Nanodrop ND-1000 Spectrophotometer (Thermo Scientific, Waltham, MA, USA) and 2100 Bioanalyzer (Agilent Technologies, Santa Clara, CA, USA) according to manufacturer’s instruction. Mutation status of these samples was initially assessed by Sanger sequencing method on AbiPrism 3100 Genetic Analyzer (Applied Biosystems, Foster City, CA, USA) using BigDye Terminator v3.1 Sequencing Kit (Applied Biosystems, Foster City, CA, USA) according to manufacturer’s instruction.

Genotyping assay of target DNA sequences, harboring the variation of interest, was performed through two asymmetric multiplex PCR implemented on standard equipment LightCycler 1.5 (Roche) to pre-optimize the PCR amplification characteristics, such as melting temperature, primers concentration and efficiency. Briefly, 12.5 μL of reactions mixture was provided comprising 0.2 μM of forward primer, 2.4 μM of reverse primer, 2.5 U of Hot Start Taq^plus^ DNA polymerase (Qiagen), 25 mM of MgCl_2_, 10 mM of deoxynucleotide triphosphates (dNTPs), 10X PCR Buffer and 20 ng/µL of genomic DNA. Quantitative PCR (qPCR) was run with the following conditions: initial denaturation for 5 min at 95 °C followed by 40 cycles of denaturation for 1 min at 94 °C, annealing for 1 min at a different temperature in a range of 58 ± 2.5 °C, and final extension for 10 min at 72 °C. Once standardized, the two multiplex PCR protocols were performed in the LoC of the In-check platform, where PCRs take place in two separate chambers microfluidically connected to microarray area. On LoC, a total volume of 11.5 μL of PCR mix was loaded with standard pipette tips into two channels inlet sealed then by specific clamps. The assembled chip was loaded onto TCS module and the PCR reactions were performed according to the optimized conditions. Upon completion of PCR, to assess the efficiency of the method and the specificity of amplicons obtained, the products were recovered by centrifugation of the chip in a 50 mL falcon tube for 2 min and analyzed by Agilent 2100 Bioanalyzer. 

Thanks to a microfluidic connection between the PCR and microarray modules, the hybridization step can be performed subsequently to the PCR amplification. The hybridization conditions to obtain better probe performances, in terms of sensitivity and specificity were optimized. In particular, the period of incubation, hybridization temperature, the composition of hybridization buffer and washing stringency were assessed. The hybridization buffer was composed of 0.1% of Tween 20, 50 mM of phosphate buffer, 0.7 M of NaCl, 2 ug/mL of Salmon sperm DNA, 2× of Denhardt’s solution, and 500 nM of the positive hybridization primer control AT683. For the integrated PCR-microarray experiment, after target amplification, 14.5 µL of hybridization buffer mix was added to each sample loaded on the chip placed onto the TCS module. The samples hybridization was performed at the following conditions: 95 °C for 3 min and 58 °C for 30 min. After the hybridization, the chip was washed into 50 mL Falcon tubes containing 20% SDS, 20× saline sodium citrate (SSC) buffer and Milli-Q water, in order to remove excess hybridization buffer and unbound target; this step was performed by centrifugation at 3000 rpm at room temperature for 2 min and dried by a second centrifugation at the same conditions. To acquire the array image, the chip was scanned with an OR. The scanning process produced a digital image revealing the distribution of hybridized target. After image acquisition, the overlapping of a virtual grid over the file allowed to delimit each probe cell and evaluate the spot quality. The hybridization reaction has led to the development of fluorescence on the chip at the site of probe binding. The mutations were, therefore, detected by lack of binding to wild-type probes, as well as by perfect match with specific probes for the mutations. 

### 2.3. Analytic Performance Studies

The efficiency and limits of the in-house developed methods on the In-Check platform for the intended analytical applications were valued analyzing the repeatability, sensibility and specificity by detection of Limit of Blank (LoB) and Limit of Detection (LoD) [32]. The validity of the method was demonstrated in laboratory experiments using clinical samples and an in silico template. To perform an in silico validation, we used a site-directed mutagenesis method [33]. In particular, a synthetic primer containing the mutation of interest (g.10571G > Tp.Gly12Val) was designed as complementary to the DNA template. PCR amplification allowed the incorporation of a point mutation into the amplicon, replacing the original sequence. A second PCR produced a fragment containing the desired mutation in sufficient quantity to be detected. To evaluate the efficiency of our method to generate the mutagenized target, we used a Real Time PCR (LightCycler by Roche Diagnostics) and Automatic Sequencing methods (AbiPrism 3100 Genetic Analyzer by Applied Biosystems). Both confirmed the incorporation of KRAS G12V mutation into the DNA template.

To assess the LoD of the array system, we performed experiments with serial dilutions of the mutagenized target starting from 10^12^ to 10^2^ copies without a pre-amplification assay. The initial concentration of template was measured by Nanodrop-1000 spectrophotometer. The LoB parameter was estimated evaluating samples without templates. Each measurement was measured in triplicate and the mean result was calculated. The assay repeatability was evaluated by use of 20 replicates of KRAS mutated and wild-type samples. Each of these samples was aliquoted into the appropriate number of individual vessels as identical replicates of the original sample. Then, each replicate was run through all steps of the assay with the same user and reagent lot. To predict the inter- and intra-assay variability of allele capture probes fluorescence intensities (F.I.), the line of the best fit for Site-Directed Mutant and clinical samples was estimated. The median signals of fluorescence intensities of allele-capture probe with the 95% of confidence intervals was considered. Briefly, for inter-assay variability a cross correlation of technical replicates was estimated, meanwhile, for intra-assay variability, a cross correlation of fluorescence intensity of identical capture probe was evaluated in the same assay. The Pearson correlation coefficient values are indicated by R^2^. P-values < 0.05 were considered significant.

## 3. Results

### 3.1. Primers Selection and PCR Optimization

**Figure 2:**
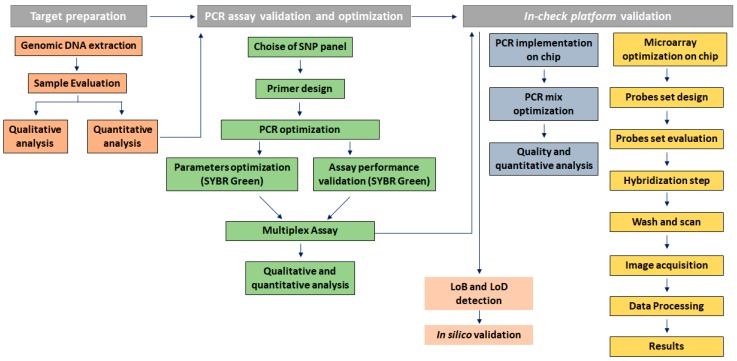
Genotyping assay process to identify KRAS point mutations by analyzing probe-gene mapping.

**Figure 3:**
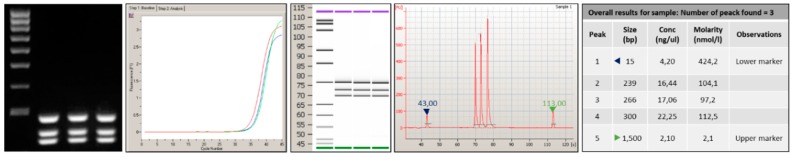
Analysis performed by 2100-Bioanalyzer system of amplicons generated after PCR amplification onto Lab-on chip. The products obtained show an appropriate size difference to allow the discrimination and a good differentiation between the exons 2, 3, and 4.

Figure 2 depicts the genotyping assay process performed to identify KRAS point mutations by analyzing probe-gene mapping. The first step has been the optimization of PCR assay to amplify and detect the specific variants in target genomic DNA. Primer set with a range of melting temperature of 58 ± 2.5 °C, GC content between 45–55% and products of different sizes were designed for the detection of a point mutation in exon 2, 3, and 4. In particular, a size range of 100 to 300 bp was chosen to minimize the likelihood of amplification of non-specific products and increase the thermocycling speed (Table 2). To implement the protocol on the In-check platform, the primers were tested in combination and two multiplex PCR were developed; the optimum annealing temperature for the primer pairs was identified as 61 °C (Table 2). The sensitivity comparison between the conventional real-time PCR and amplification on In-Check was evaluated by the capillary lab-on-a-chip electrophoresis 2100-Bioanalyzer system. The optimal combination of annealing temperature, primers mix and PCR buffer allowed to obtain by In-check assay, the highly specific amplification products. As displayed in Figure 3, the generated amplicons show an appropriate size difference to allow the discrimination of the fragments by electrophoresis and a good differentiation between the exons 2, 3, and 4. Consequently, the assay described has been properly developed and optimized for the intended purpose.

### 3.2. Probes Set Selection and Hybridization Assay Evaluation

The probe selection was based on criteria described in the Materials and Methods. For each target sequences selected, two different oligonucleotide probe sets were designed to discriminate wild-type and mutant sequences; each probe contains an appropriate modification that allows the covalent attachment of the oligonucleotide to the chip array surface. Probes generated had a length of 18–20 bases, a melting temperature in the range of 58–62 °C, to facilitate uniform hybridization conditions and do not contain self-complementary regions (Figure 1). 

**Figure 4:**
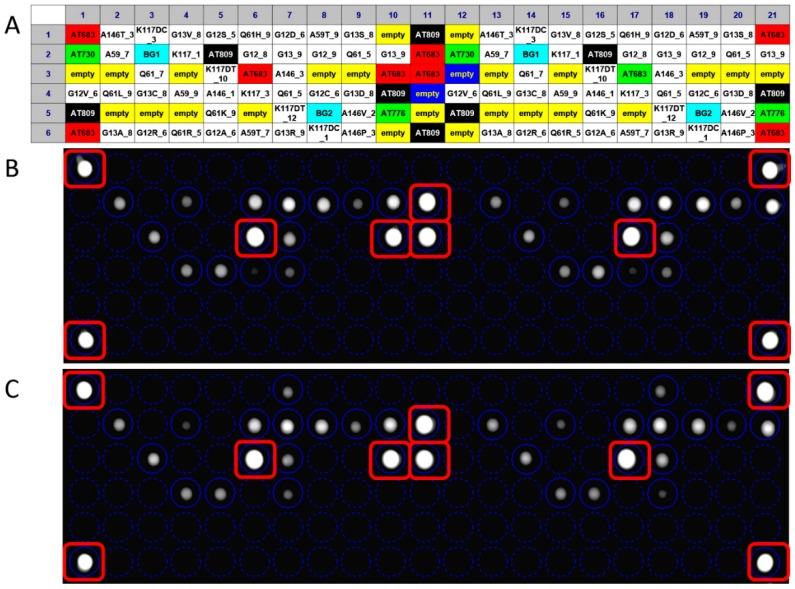
Result of a fully integrated experiment; the panel (**A**) shows the layout of capture probes spotted on the chip array. The overlapping of the virtual grid allows to select each probe cell and the identification of probe spotted. Panel (**B**): Genotyping assay of wild type KRAS clinical sample; Panel (**C**): Genotyping assay of G12D mutated KRAS clinical sample.

Concerning the competitive hybridization process, the labeled target sequence obtained after the PCR amplification was captured on the chip surface from probe match, producing a fluorescence signal. The fluorescence derives from gene-specific binding revealing the sequence perfectly complementary to the query sequence. The hybridization signals were analyzed by the high-resolution OR. The digital image was analyzed by dedicated software and the poor-quality spots were flagged. The genotype discrimination may be determined by perfect hybridization with only wild-type-specific or mutated-specific capture probes and a partial hybridization of both allele specific capture probes: a perfect match defines a homozygous genotype, a partial hybridization defines a heterozygous genotype. In the example shown in Figure 4, the analysis of wild-type KRAS clinical sample generated fluorescence signals only in corresponding to wild-type-specific capture probes (Figure 4B), as well as the binding of the target sequence with a complementary probe gave a strong positive signal corresponding to G12D-specific and wild-type-specific capture probes (Figure 4C). The allelic discrimination performed by In-check assay has confirmed the samples genotypes, classifying the first sample as heterozygous mutated genotype. The fluorescence signal was also generated from control probes used to normalize the hybridization signals, remove the noise and measure the spot array quality [34]. The data obtained showed that the hybridization conditions used were appropriate to obtain minimum background noise and signal specificity. The method performed on the In-check platform allowed the unambiguous and correct genotyping of the sample analyzed providing higher levels of discrimination. 

### 3.3. Analytical Sensitivity and Specificity

**Figure 5:**
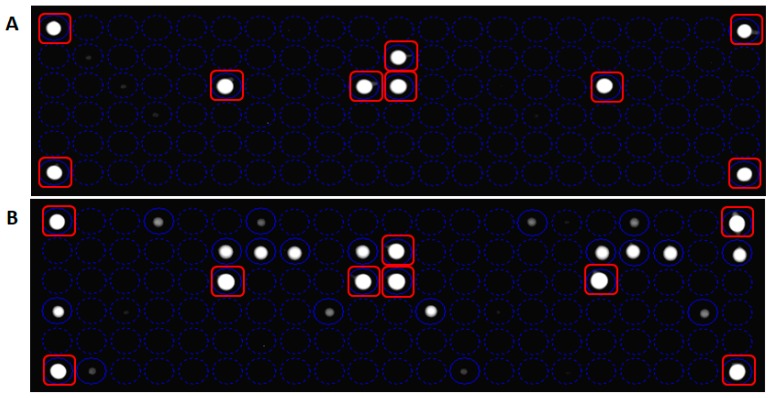
Panel (**A**): Limit of Blank (LoB) detection by measurement of blank sample. The fluorescent signal is related to control probes. Panel (**B**): Limit of Detection (LoD) detection related to mutagenized G12V. The In-check platform is able to measure an analytical signal in a sample containing 10^10^ copies of the target. Hybridization controls probes are marked with red squares.

The measure of LoB, a parameter particularly crucial in a test where the results are reported as present or absent, has demonstrated the sensibility of In-Check platform in the detection of non-analytical signal when sample without analyte was analyzed (Figure 5A). The LoD value of the array system, defined as the lowest quantity of analyte that can be reliably detected in a sample, was obtained performing a direct hybridization on the chip array, without a pre-amplification step. As shown in Figure 5B, data analysis related to mutagenized G12V template reveals as the In-check platform was able to measure an analytical signal in a sample containing 10^10^ copies of target. 

**Figure 6:**
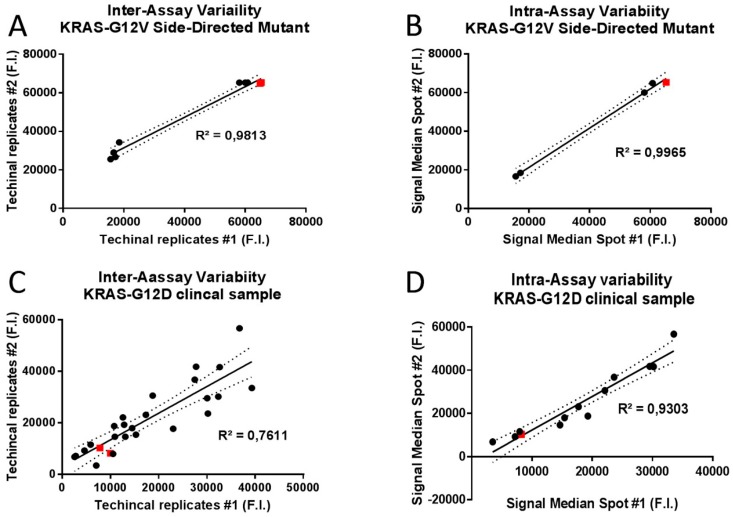
Scatterplots and linear regression analysis for inter and intra-assay variability of allele capture probes fluorescence intensities (F.I.) in two randomly selected replicates. Representative scatterplots of 10^10^ copies of KRAS-G12V Side-Directed Mutant product were generated to graphically display the linearity of pairwise relationships between technical replicates in inter-assay variability (R^2^ = 0.9813; *p* < 0.0001; panel **A**) and between median fluorescence intensities of identical capture probe in intra-assay variability (R^2^ = 0.9965; *p* < 0.0001; panel **B**). Representative scatterplots of the KRAS-G12D clinical sample were generated to graphically display the linearity of pairwise relationships between technical replicates in inter-assay variability (R^2^ = 0.7611; *p* < 0.0001; panel **C**) and between median fluorescence intensities of identical capture probe in intra-assay variability (R^2^ = 0.9303; *p* < 0.0001; panel **D**). Best fits in solid lines. 95% confidence intervals in dashed lines. Red squares display the position of allele specific capture probes on the best fit lines.

To predict the inter- and intra-assay variability of KRAS-complementary spot hybridization, the R^2^ Pearson correlation coefficient based on cross correlation between the median fluorescent intensity of technical replicates and identical capture probe in the same assay respectively. Raw expression data for 10^10^ copies of KRAS-G12V Side-directed Mutagenesis product and KRAS-G12D clinical sample hybridization assays were normalized and Pearson’s correlation coefficients were calculated for the data sets of hybridization signal intensities. High correlation coefficients were obtained both for inter- (R^2^ = 0.9813; *p* < 0.0001; Figure 6A) and intra-assay variability (R^2^ = 0.9965; *p* < 0.0001; Figure 6B) of KRAS-G12V Side-directed Mutagenesis product hybridization. Similarly high correlation coefficients were obtained also for intra-assay variability of KRAS-G12D clinical sample (R^2^ = 0.9303; *p* < 0.0001; Figure 6D); meanwhile lower correlation coefficients were obtained for inter-assay variability of KRAS-G12D clinical sample (R^2^ = 0.7611; *p* < 0.0001; Figure 6C).

These results indicate an almost perfect correlation between different technical experiments and between hybridization signal intensities of identical allele-capture probes in the same assay, underlining the extremely high precision level of In-Check platform results.

## 4. Discussion

In this manuscript, we describe the use of the In-check platform, a miniaturized silicon device easily transferable from industry to the clinic for highly sensitive and reliable applications in the molecular diagnostic area [18]. Based on MEMs (micro electromechanical systems) technology, the revolution that makes this device the most promising technology for genotype analysis is the microfluidic integration of PCR amplification and low-density microarray hybridization on the same chip, making possible the realization of complete systems-on-a-chip. Key enabling features of this diagnostic platform are the reduced time to results, more information in one test, disease-specific or therapy specific patterns, identification and mutations analysis available simultaneously for drug resistance and drug susceptibility. Currently, the In-check platform is used to detect, identify and differentiate several biological materials such as influenza virus subtypes, multiple foodborne and avian pathogens, different mycobacterium strains or biological weapons [35,36]. In this work, the In-check platform was validated to assess the mutational status of KRAS oncogene, the main factor responsible for the development of different cancers, in particular CRC, giving them resistance to anti-EGFR therapies. The results obtained demonstrated the great efficiency of the developed method in terms of sensibility and specificity, rendering the In-check platform “fit for purpose” and suitable for a multitude of applications ranging from diagnostic to clinical, industrial and research areas [37]. This platform has proved easy to use, less time consuming and cheaper than conventional methods. Moreover, the proposed diagnostic approach used to select patients for targeted therapy with the anti-EGFR antibody, can improve molecular pathological epidemiology (MPE) enhancing molecular pathologic signatures contributing to precision medicine for personalized prevention and treatment [38,39].

## 5. Conclusions

The genomics revolution, fueled by advances in biotechnology tools, offers new ideas to achieve, analyze data and better understand genetic variation. New approaches aimed to obtain a better classification of patients, and personal genetics and -omic profiles can contribute to an effective personalized medicine and clinical management of many genetic disorders. The concept of personalized medicine is not new: it has always been observed that patients respond differently to medical interventions [40]. The news is the development of new promising technologies able to predict the answered to a medical therapy and its effects [41]. Advances in micro/nanotechnology are pioneering the development of new generation methodologies that are highly sensitive, simple, reliable and rapid, and useful in biology and medical research for diagnosis and screening of several human diseases, such as neurodegenerative, oncological, or metabolic diseases [42,43]. The present work demonstrates that the In-Check platform can be used efficiently for nucleic acids analysis, allowing the systematic determination of the polymorphic sequences that vary to the level of a single nucleotide.

## Figures and Tables

**Table 1:** KRAS point mutations panel detected by array-based platform.

Exon 2	**rs121913530**
	10570G>T/G>C/G>A-->Gly12Cys/GLY12ARG/Gly12Ser
	LUNG CANCER, SQUAMOUS CELL, SOMATIC
	BLADDER CANCER, SOMATIC, INCLUDED
	**rs121913529**
	10571G>C/G>T/G>A--> Gly12Ala/GLY12Val/Gly12Asp
	LUNG CANCER, SQUAMOUS CELL, SOMATIC
	BLADDER CANCER, SOMATIC, INCLUDED
	**rs121913535**
	10573G>T/G>C/G>A --> Gly13Cys/Gly13Arg/Gly13Ser
	BREAST ADENOCARCINOMA, SOMATIC
	**rs112445441**
	10574G>A/G>C/G>T --> Gly13Ala/Gly13Asp/Gly13Val
	BREAST ADENOCARCINOMA, SOMATIC
Exon 3	**rs121913528**
	28572G>A/Ala59Thr
	BLADDER CANCER, TRANSITIONAL CELL, SOMATIC
	**rs121913238**
	28578C>A-->Gln61Lys
	COLORECTAL CANCER
	**rs121913240**
	28579A>T/A>G-->Gln61Leu/Gln>Arg
	NON-SMALL CELL LUNG CANCER
	**rs17851045**
	28580A>C/A>T-->Gln61His
	COLORECTAL CANCER
Exon 4	**rs770248150**
	30208A>C/A>T-->Lys117Asp)
	COLORECTAL CANCER
	**rs121913527**
	30293G>C/G>A-->Ala146Pro/Ala146Thr
	COLORECTAL CANCER
	**rs1057519725**
	30294C>T-->Ala146Val
	COLORECTAL CANCER

**Table:** 

Name	Primer F	Primer R	Product Size	Basic Tm
KRAS exon 2	ACTGGTGGAGTATTTGATAGTGTAT	AGAATGGTCCTGCACCAGTAA	249	52 °C
KRAS exon 3	AGGTGCACTGTAATAATCCAGACT	AACCCACCTATAATGGTGAATATCT	228	53 °C
KRAS exon 4	AAGGACTCTGAAGATGTACCTATG	AGAAGCAATGCCCTCTCAAG	293	53 °C

**Table:** 

Reagents	Volume (µL)	Final Concentration
Forward Primer 10 µM	0.5	0.2 µM
Reverse Primer 10 µM	0.5	0.2 µM
HotStart Taq plus DNA Polymerase	0.4	2.5 U
Cl_2_Mg 25 mM	0.2	0.5 mM
dNTPs Solution Mix 10 mM each	0.5	4 mM each
Buffer 10X	2.5	
Genomic DNA	5	20 ng/µL
DNase Free Water	1	/
Total Volume	12.5	

**Table:** 

Cycle Steps	Temp.	Time	Number of Cycles
Initial Denaturation	95°	900 s	1
Denaturation	94°	60 s	35
Annealing	61°	60 s	
Extension	72°	60 s	
Final Extension	72°	600 s	1
